# The Effect of Contact Angles and Capillary Dimensions on the Burst Frequency of Super Hydrophilic and Hydrophilic Centrifugal Microfluidic Platforms, a CFD Study

**DOI:** 10.1371/journal.pone.0073002

**Published:** 2013-09-12

**Authors:** Amin Kazemzadeh, Poo Ganesan, Fatimah Ibrahim, Shuisheng He, Marc J. Madou

**Affiliations:** 1 Department of Mechanical Engineering, University of Malaya, Kuala Lumpur, Malaysia; 2 Medical Informatics & Biological Micro-electro-mechanical Systems (MIMEMS) Specialized Laboratory, Department of Biomedical Engineering, University of Malaya, Kuala Lumpur, Malaysia; 3 Department of Mechanical Engineering, University of Sheffield, Sheffield, United Kingdom; 4 Department of Biomedical Engineering, University of California Irvine, Irvine, California, United States of America; 5 Department of Mechanical and Aerospace Engineering, University of California Irvine, Irvine, California, United States of America; 6 Ulsan National Institute of Science and Technology (UNIST), World Class University (WCU), Ulsan, South Korea; Texas A&M University, United States of America

## Abstract

This paper employs the volume of fluid (VOF) method to numerically investigate the effect of the width, height, and contact angles on burst frequencies of super hydrophilic and hydrophilic capillary valves in centrifugal microfluidic systems. Existing experimental results in the literature have been used to validate the implementation of the numerical method. The performance of capillary valves in the rectangular and the circular microfluidic structures on super hydrophilic centrifugal microfluidic platforms is studied. The numerical results are also compared with the existing theoretical models and the differences are discussed. Our experimental and computed results show a minimum burst frequency occurring at square capillaries and this result is useful for designing and developing more sophisticated networks of capillary valves. It also predicts that in super hydrophilic microfluidics, the fluid leaks consistently from the capillary valve at low pressures which can disrupt the biomedical procedures in centrifugal microfluidic platforms.

## Introduction

Conventional clinical diagnostic tasks such as Enzyme-Linked Immunosorbent Assays (ELISAs) consist of a series of sequenced procedures carried out by skillful operators until the final analytical results are obtained [1]–[4]. These processes are time consuming and highly dependent on the skills of trained and experienced operators. Alternatively, centrifugal microfluidic platforms have been proven to be an attractive candidate for applications of various biomedical/biotechnology procedures such as blood plasma separation, disease screening, drug testing, cellular and chemical analysis, etc. [5]–[7]. Centrifugal microfluidic platforms do not influence the important physicochemical properties of fluids such as pH or ionic strength that facilitates the flow control of different types of aqueous solutions (blood, mucus, urine, milk) [4]. They are fast, cost efficient and automated that reduce human errors especially in the diagnosis of diseases. To date, many complex analysis procedures have been demonstrated on a single disk in order to develop sample-to-answer systems or micro total analysis systems (µTAS) [4], [6], [7].

Centrifugal microfluidic platform requires minimal external instrumentation to propel and manipulate fluids and its fabrication is a well-established art. It is placed on a rotating system and uses centrifugal force to drive and manipulate fluids. The rotational speed of the disk, the exact shape of the chambers and fluidic conduits together with their positions on the disk are used for flow control and flow sequencing [4], [8]–[10]. Centrifugal microfluidic platforms consist of a specially designed sequence of micro channels, micro valves and chambers. Microvalves on a centrifugal microfluidic platforms can be categorized as passive valves or active valves: the former only need a change in rotation speed (rpm) to be actuated but the latter require a force different than the centrifugal force e.g., heat in the case of paraffin wax plugs [4]. Using passive valves in centrifugal microfluidic platforms is popular and the valves are often classified as non-mechanical and mechanical [1], [4], [9], [11]–[15]. Mechanical passive valves usually consist of flaps [16], [17], membranes [14], [18], [19], balls [20], [21] and so on; while non-mechanical passive valves, such as capillary valves are often use geometry properties [9], [22], [23] or surface properties in microchannels [24].

Flow in capillary valves is controlled by the interaction between the centrifugal forces and the capillary forces. The fluid advancement in the capillary is stopped when a capillary meets a sudden change in its geometry. The maximum rotational speed required for overcoming that pressure barrier is referred to as the burst frequency. The effects of capillary dimension, expansion angle and capillary shapes on burst frequencies in hydrophilic and hydrophobic microstructures have been thoroughly studied [8], [25]. For instance Chen et al. [25] have studied the effect of the expansion angle and the capillary dimension on the burst frequency of the microstructures with contact angle of 68°. A number of theoretical models to calculate burst pressure in a centrifugal microfluidic platform have been presented in the literature [8], [22], [25]–[29]. These theoretical models are most reliable for predicting burst frequencies in capillaries made of PMMA where the contact angle for DI-water is about 60°–70° because they have been experimentally tested on centrifugal microfluidic platforms made from such materials. Experiments conducted by He et al. [26] show that the analytical models can also be used well in centrifugal microfluidic platforms made of super hydrophobic materials where, the contact angle of DI-water can be up to 165°. However, theses equations are not supported by experiments when they are used to calculate the burst frequency in super hydrophilic and less hydrophobic centrifugal microfluidic platforms.

This paper focuses on flow behaviour in super hydrophilic to less hydrophobic centrifugal microfluidic platforms and attempts to provide a detail view of capillary valves function in such microfluidic platforms. Various capillary structures with the static contact angle range 20° to 90° are investigated. The capillary dimensions are varied from 150 µm to 450 µm to study the effect of dimensions and the contact angle on the burst frequency. The volume of fluid method within version 13.0 of commercial code of ANSYS-Fluent is used for solving the governing equations. In order to validate the implementation of the numerical model our experimental data and existing experimental data in the literature are compared to the present computed data.

## Methodology

### 2.1 Governing equations

#### 2.1.1 Numerical

**Figure 1:**
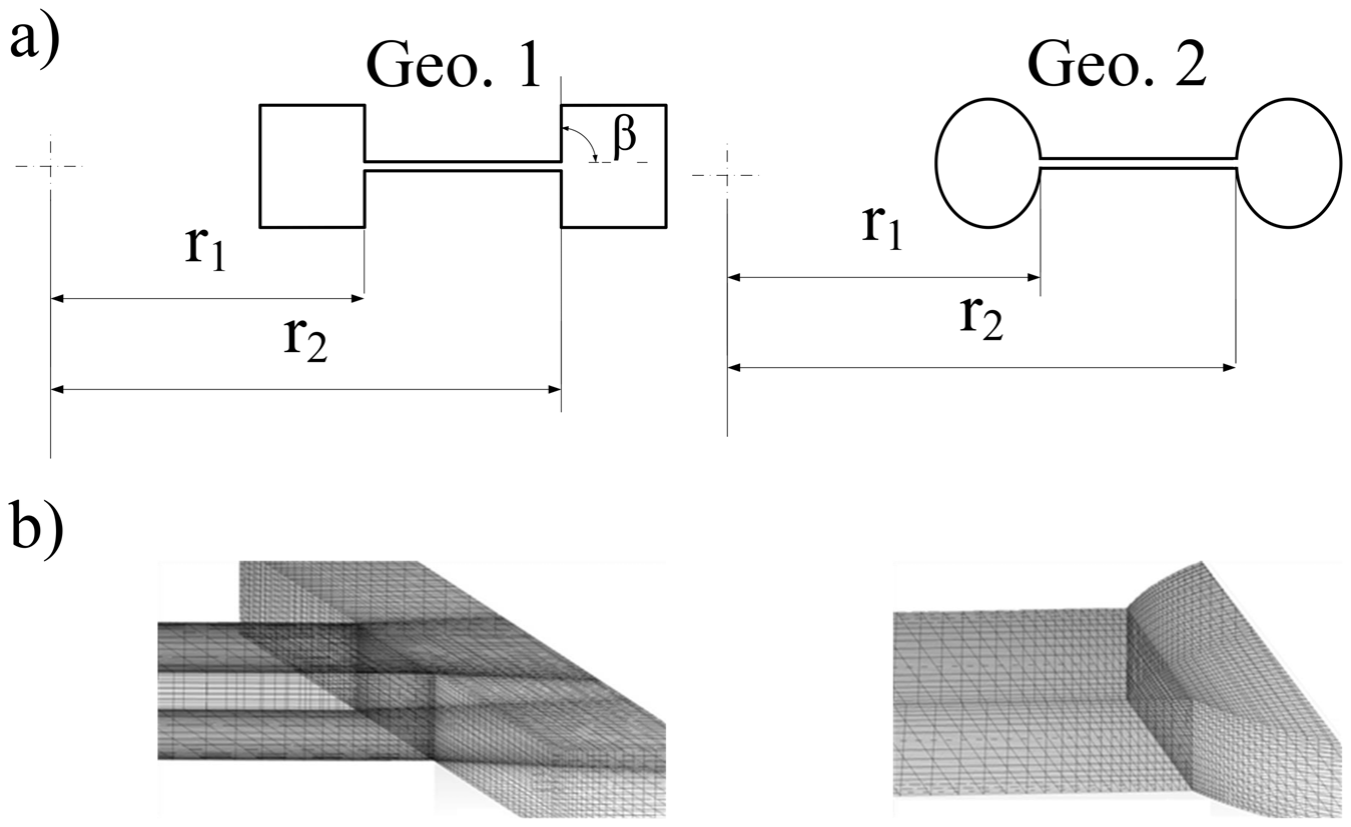
a) Top view of Geo. 1 and Geo. 2 b) Computational mesh adjacent to the outlet of capillary channel of Geo 1 and Geo 2.

We used the volume of fluid (VOF) method within the commercial ANSYS-Fluent CFD package, version 13.1 to predict the burst frequency in Geo. 1 and Geo. 2 (see Fig. 1). This method is computationally inexpensive and provides reliable results for this type of study [30]–[32]. In the VOF algorithm, the dynamic contact angle i.e., the angle formed between the moving liquid interface and the solid interface at three-phase contact line [33],[34], is automatically calculated as part of the solution via finite volume method from the basic equilibrium of forces in the numerical method [30], [35], [36]. The VOF algorithm computes the macroscopic effect of surface tension by tracking the contact line and does not impose a constant contact angle at the surface. In other words, the predefined contact angle is continuously changing based on the velocity and the direction of the contact line [37]. However, since the contact line tracked in the VOF algorithm is based on the macroscopic level of interaction between the three-phase, it is necessary to ensure the viability of the algorithm in the simulation of capillary flows. Saha et al. [38] have used the VOF method to investigate the fluid flow in capillary channel made of PDMS using both the static and the dynamic contact angles. The latter was calculated using eight different types of theoretical models from literature e.g., Blake, Bracke, Newman and Shikhmurzaev [39], [40] and incorporated into Fluent via a User Defined Function (UDF). However, no significant difference was found in results due to the use of the two types of contact angles. The study was carried out for contact angles of 0°, 36° and 72° and various surface tensions and viscosities. Therefore, the physics occurring in the nano-scale level at the three-phase contact line can be addressed quite well in the VOF method [30], [35]–[37], [41]. Note that, the cases studied in Saha et al. [38] are spontaneous wetting cases. Forced wetting problems e.g., flow in centrifugal microfluidic platforms have the same underlying mechanisms and are described in an equivalent way to a spontaneous wetting problem since they are instances of moving contact lines [38], [42].

In the VOF method, the position of the interface between the fluids of interest is tracked in a fixed Eulerian mesh domain. A single set of Navier-Stokes equations is solved for the computational domain and the volume fraction of each fluid is tracked by using an additional transport equation. The volume fraction (a) in each cell in the computational domain is between 0 and 1. For control volume, the sum of the volume fraction of all phases is set to unity. Therefore, any given cell represents either a mixture of phases (0<a<1) or a pure phase (a = 1) flow. The continuity and momentum equations for laminar, incompressible, Newtonian, and isothermal flow employed in the current study are as follows [12]:
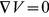
(1)

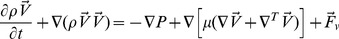
(2)where, V, P, t, F_v_, ρ and µ are the velocity of the mixture, pressure, time, volumetric forces, density and viscosity, respectively. The continuity equation of a multiphase immiscible flow in Fluent is solved solely for the secondary phase q_th_, which has the following form:
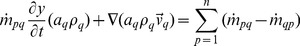
(3)where, m˙_pq_ is the mass transfer from phase p to phase q and m˙_pq_ is the mass transfer in the reverse direction. The primary-phase volume fraction is computed using the following constraint:
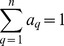
(4)


The average values of variables and properties of the mixture are defined based on the volume fraction of each phase at a given location [32]. For instance, the average values of density and viscosity of the mixture in a computational cell are: 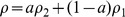
(5)

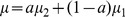
(6)


The continuous surface force model (CFS) is used to reformulate surface tension into an equivalent body force [36]. For a two-phase system, the volumetric force due to surface tension at the interface between phases 1 and 2 is given as: 
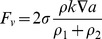
(7)


where, ρ is the volume-averaged density computed using Eq. 5, ρ_1_, ρ_2_ are the density of phase 1 and 2, respectively. According to the CSF model, the surface curvature k is computed from local gradients in the surface normal to the interface, which is given as: 
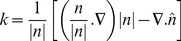
(8)


where n = ∇a is the normal vector. Wall adhesion is included in the model through the contact angle:
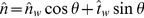
(9)


where nˆ is the unit vector normal to the surface, 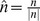, nˆ_w_, tˆ_w_ represents the unit vector normal and tangent to the wall, respectively.

#### 2.1.2 Theoretical models

A simple equation introduced by Zeng et al. [27] that only take the capillary width into account and two sophisticated model from Chen et al. [25] and He et al. [26] are adopted for comparison with the numerical results. The first model uses capillary pressure difference in the liquid-air interface at the meniscus derived from the Young-Laplace equation. By minimization of the energy and from the equilibrium of the forces involved, Zeng et al. [27] presented the capillary pressure formulation as: 
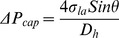
(10)


where, D_h_ is the capillary hydraulic diameter, θ is the static contact angle and σ_la_ is the liquid air surface tension. In Chen et al. [25] and He et al. [26] models other parameters such as the expansion angle and capillary height are taken into account. In the former all surfaces of the valve are assumed to have the same contact angles of θ:
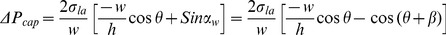
(11)


where w and h are the width and the height of the capillary channel, β is the expansion angle. The expression is developed based on two stages of fluid advancement in a capillary which can be summarized as: a) prior to the valve, b) at the valve (transition period). At the first stage, pressure in the liquid can be derived from either the Young-Laplace equation or the change of total interfacial energy of the solid–liquid–air system by using the static contact angle. The second stage considers the influence of the changes in advancing contact angle and the changes of the meniscus shape from a concave to a flat and from the flat to a convex meniscus. At this stage it is assumed that the meniscus arcs in the width of the capillary (with angle of α_w_) and in the height (with angle of α_h_) vary equally until the meniscus arc in height direction stops changing. The meniscus arc in the width of capillary continues changing until the liquid bursts into the expanded volume (where α_w_ = π/2- θ-β).

In the last model, in addition to height and expansion angle different contact angles associated with the capillary surfaces are taken into account: 
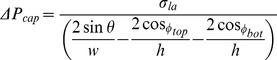
(12)


where, φ_top_ and φ_bot_ are the contact angles of a fluid at the top and bottom substrate respectively. A schematic view of the side and top of the fluid motion in a capillary made of different materials presented in Leu et al. [23] shows that the meniscus is dragged forward at the top and bottom surfaces because of the wetting forces. At the side surfaces, however, the liquid is held back because of its surface tension forces. The burst frequency is calculated by equalizing the centrifugal and the resultant of the surface tension forces.

### 2.2 Boundary conditions and Numerical method

Two common types of microstructures, Geo. 1 and Geo. 2 (see Fig. 1a) are used to study the effect of contact angles and capillary dimensions on burst frequency. Geo. 1 features square chambers and Geo. 2 circular chambers. In both structures, a capillary channel is located between the two chambers and fluid flows from the left chamber to the right one. The left chamber (close to the CD center) is referred to as the entry chamber and the right one as the outlet chamber. The distance of the capillary valve from the center of the rotation (r_2_), which is also the center of the disk, for Geo. 1 and Geo. 2 is 31.5 mm and 43.57 mm, respectively. The surface tension of water is set at 0.072 N/m. These parameters in addition to the value of advancing and equilibrium contact angles are the same as those used in the experiments carried out by Chen et al. [25] and He et al. [26] and will be used for validation of our numerical data. The contact angle at solid walls has been specified according to the cases listed in Table 1. The computational domains of Geo. 1 and Geo. 2 are set to rotate clockwise using a single rotational frame (SRF) [32] in order to propel the fluid from the left chamber to the right chamber through the capillary channel. The rotational speed starts from a low frequency, i.e. 25rpm and it is gradually increased at intervals of 50rpm. All the solid boundaries of the domain are treated as walls with zero slip velocity. A zero pressure gradient is assumed from fluid entrance on the left to fluid exit on the right. The left chamber is filled with a sufficient volume of water such that the water consistently occupies the capillary channel until it bursts and fills some volume in the right chamber. In order to reduce the computational time simulations begin when half of the capillary is filled with the water. In addition, we have investigated a case where left chamber is filled with water allowing it to flow from the left chamber into the capillary channel before bursting to the right chamber. However, our numerical data show that there is no difference in the results between the above two types of initial conditions.

**Table 1:** Details of simulation Cases 1–36.

Num.	Aspect ratios	θ°	Geo.	Remarks
Cases	h/w			
1–4	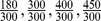	68	1	These cases are used for
	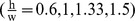			validation and are the
				same to that used in Chen
				et al. [25].
5–10	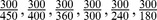	68	1	These cases are used to
	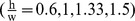			study the effect of
				capillary dimensions on
				burst frequency and h/w is
				the inverse of that used in
				Chen et al. [25].
11–18	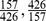 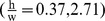	20:10:80	2	These cases are used to
,19–26		and 93		study the effect of θ on
27–31	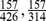 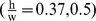	45:10:85	1	burst frequency, where,
,32–36				h/w = 157/426 and
				h/w = 426/157 is the same
				to and the inverse of that
				used in He et al. [26]
				respectively.

The Pressure Implicit Split Operators (PISO) method, which uses the splitting of operations in the solution of the discretized momentum and pressure equations is used for coupling of pressure and velocity [43]. The convection terms are spatially discretized using a second order upwind discretization method, which is an advanced finite difference scheme fully accounting for surface tension and wall adhesion forces. The body-force-weighted interpolation scheme is used in order to take into account the explicit body forces (e.g., Coriolis, centrifugal, etc.). Zonal discretization with a compressive slope limiter was set in order to have a sharper interface. An under-relaxation factor of 0.25 is used in the calculation of the pressure, density, body forces, and momentum and volume fraction. This factor reduces the rate of solution changes during the iteration to stabilize the convergence behavior of the momentum and continuity equations. The equations were solved using the unsteady model in Fluent with a time step of 0.00001s to 0.00005s for various cases of the current study. The relative error between two successive iterations was specified by using a convergence criterion of 1×10^−6^.

### 2.3 Mesh

The mesh used was based on quad grids with an element size of 0.01 mm. Grids in the area of the waterfront and the edges adjacent to expansion areas i.e., at the water outlet to the right chamber were refined to smaller element size of 0.005 mm. Mesh dependency tests were carried out for each case and the meshes eventually used were justified by the quality of the results. For instance, increasing the original mesh by 100% to obtain finer grids does not give any significant difference for the burst frequency (<1.3%). The quality of the grids used for Geo. 1 and Geo. 2 at the outlet of the right chamber is shown in Fig. 1b. The total number of cells in Geo. 1 and Geo. 2 is 437,980 and 314,852, respectively.

### 2.4 Simulation Cases

Forty-four simulation cases were created from Geo. 1 and Geo. 2 by varying the ratio of height/width (aspect ratio) and the contact angle (θ°). The range of these parameters, as listed in Table 1, represents a large sample of centrifugal microfluidic platforms scenarios. Simulation cases 1–4, 11, 32 and 37–44 are used to validate the implementation of the VOF method. These cases coincide with the experimental investigations from Chen et al. [25], He et al. [26] and Gliere et al. [44] and consist of various contact angles and capillary dimensions. Cases 5–10 are used to study the effect of the height and width dimensions of the capillary channel on the burst frequency. Here one of the dimensions is varied from 180 µm to 450 µm while the other dimension and the contact angle are kept constant at 300 µm and 68°, respectively. Cases 11–36 are used to study the effect of varying contact angles (from 20° to 90°) on the burst frequency using Geo. 1 and Geo 2.

### 2.5 Experimental set up

**Figure 2:**
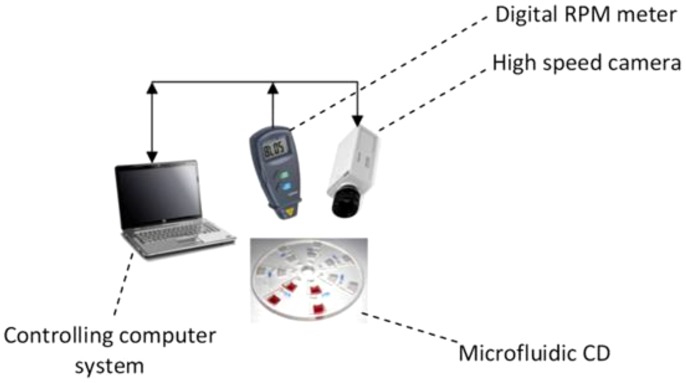
Experimental setup: controlling computer system connected to high speed camera and a digital rpm meter.

The rectangular microstructures were fabricated using a Computer Numerical Control (CNC) machine (model VISION 2525, by Vision Engraving and Routing Systems, USA). The micro structures were engraved on compact disc-like platforms layer made of a 2 mm thick Polymethyl methacrylate (PMMA) and bonded by Pressure Sensitive Adhesive (PSA) material (by FLEXcon, USA) to a 2 mm PMMA layer with venting holes cut through. A cutter plotter (model PUMA II, by GCC, Taiwan) was used to cut the microfluidic design in the PSA layers corresponding to the design of the PMMA layer. A custom-made system consists of a digital disk Spin Test System, laser sensor and a high speed camera was used to perform the experiments (see fig. 2).

## Results and Discussion

### 3.1 Validation

Several experimental studies [25], [26], [44]have been used in order to validate our numerical results of various contact angles; i.e., surfaces with the contact angles less than 40°, between 40° and 60° and from 60° to 90° which are referred to as superhydrophilic, hydrophilic and less hydrophilic surfaces, respectively. At first the experimental data from Chen et al. [25] and He et al. [26] is used to validate our numerical results for less hydrophilic cases i.e. contact angle 68°, 70° and 93°. Chen et al. [25] used rectangular microfluidic structures with an expansion angle of 90° which were fabricated of PMMA (polymethylmethacrylate) material using CNC machine. The fluid used in the test was DI-water containing a small amount of red ink. On the other hand, He et al. [26] used PMMA circular based microstructures, which were manufactured using a microinjection moulding technique. The CYTOP-coated polyaniline nanofibers were used to increase the contact angle of PMMA to 93°. The fluid used in the test was DI-water containing red food dye. The sizes and locations of the microstructures used in our numerical model for validation are set to be equal to that used in the experiment investigations, which are listed in Table 2. For the same contact angles i.e., 68, 70°, 93° and water, as the test liquid, our results are in good agreement with the measurements reported in Chen et al. [25] and He et al. [26].

**Table 2:** Comparison between the numerical and experimental burst frequencies with dimensions and positions of the capillary valves on the disk.

Case	rˆ	σ_la_	θ°	width	depth	Exp. burst	Theo. burst	Num. burst	Error	Exp.
no.	(mm)	(N/m)		(µm)	(µm)	(rpm)	(rpm)	(rpm)	(%)	Ref.
1	29.25	0.072	68	300	180	284	318	250–300	3.17	[25]
2	29.25	0.072	68	300	300	390	430	350–400	3.85	[25]
3	29.25	0.072	68	300	400	418	465	375–425	4.31	[25]
4	29.25	0.072	68	300	450	439	476	425–475	2.50	[25]
16	41.51	0.072	70	426	157	216–270	426	225–275	2.89	[26]
18	41.51	0.072	93	426	157	302–352	331	300–350	0.66	[26]

**Figure 3:**
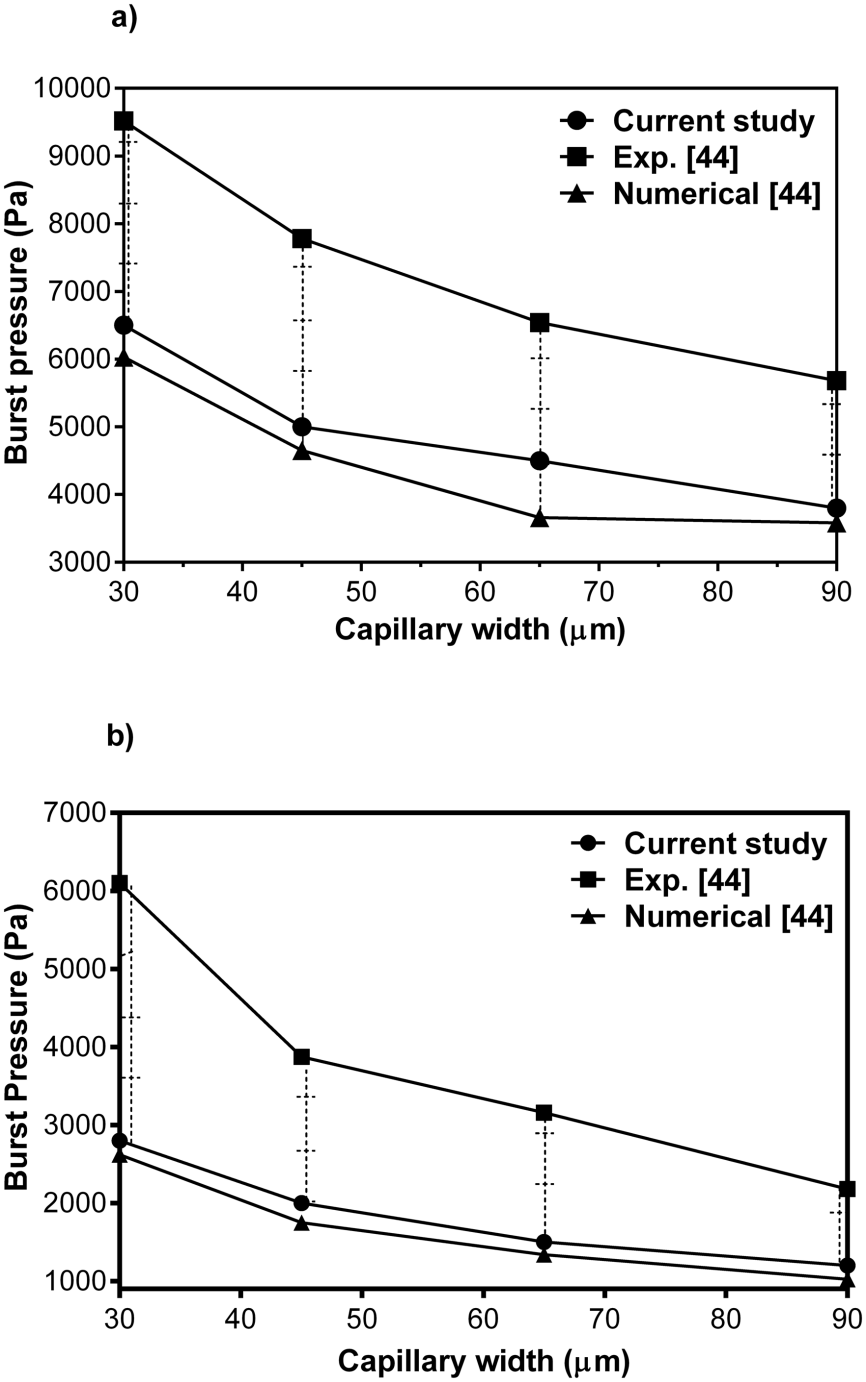
Comparison between the present study and experimental and numerical data from Gliere et al. [44], a) for DI-water (surface tension of 0.072 N/m) and b) biological buffer (surface tension of 0.03 N/m).

In the second step, the burst pressure measurements for rectangular microstructures by Gliere et al. [44] are used to further validate our numerical results for the hydrophilic and the superhydrophilic cases. In that study, silicon wafer microstructures were fabricated using a single deep etching process [45] and sealed with a PDMS substrate. DI-water with a surface tension of 0.072 N/m and a biological buffer solution with a surface tension of 0.03 N/m were used in the test. The contact angle of DI-water on the silicon wafer and PDMS are 60° and 80° and that of the biological buffer is 35° and 75°, respectively. Fig. 3 shows a comparison between our numerical results and the numerical and measured data from Gliere et al. [44]. The numerical results are in good agreement with measured and simulation data reported in Gliere et al. [44] especially for DI-water (Fig. 3a). The large deviation between our numerical data and experimental data of Gliere et al. [34] for biological buffer (Fig. 3b) may be due to an alteration of the surface tension in the experiment which causes the increase of burst pressure in comparison with the numerical results. This deviation can also be observed in the numerical data reported by Gliere et al. [34]. In fact, in the experiment, during the expansion of a meniscus the surfactant concentration reduces to a value lower than the equilibrium concentration which causes the increase in surface tension value. In addition during the relaxation time of the surface tension and surfactant the actual surface tension of the buffer is larger than the equilibrium surface tension. However, the surface concentration value gradually increases back to its equilibrium value [44], [46].

### 3.2 Flow sequence in a super hydrophilic capillary

**Figure 4:**
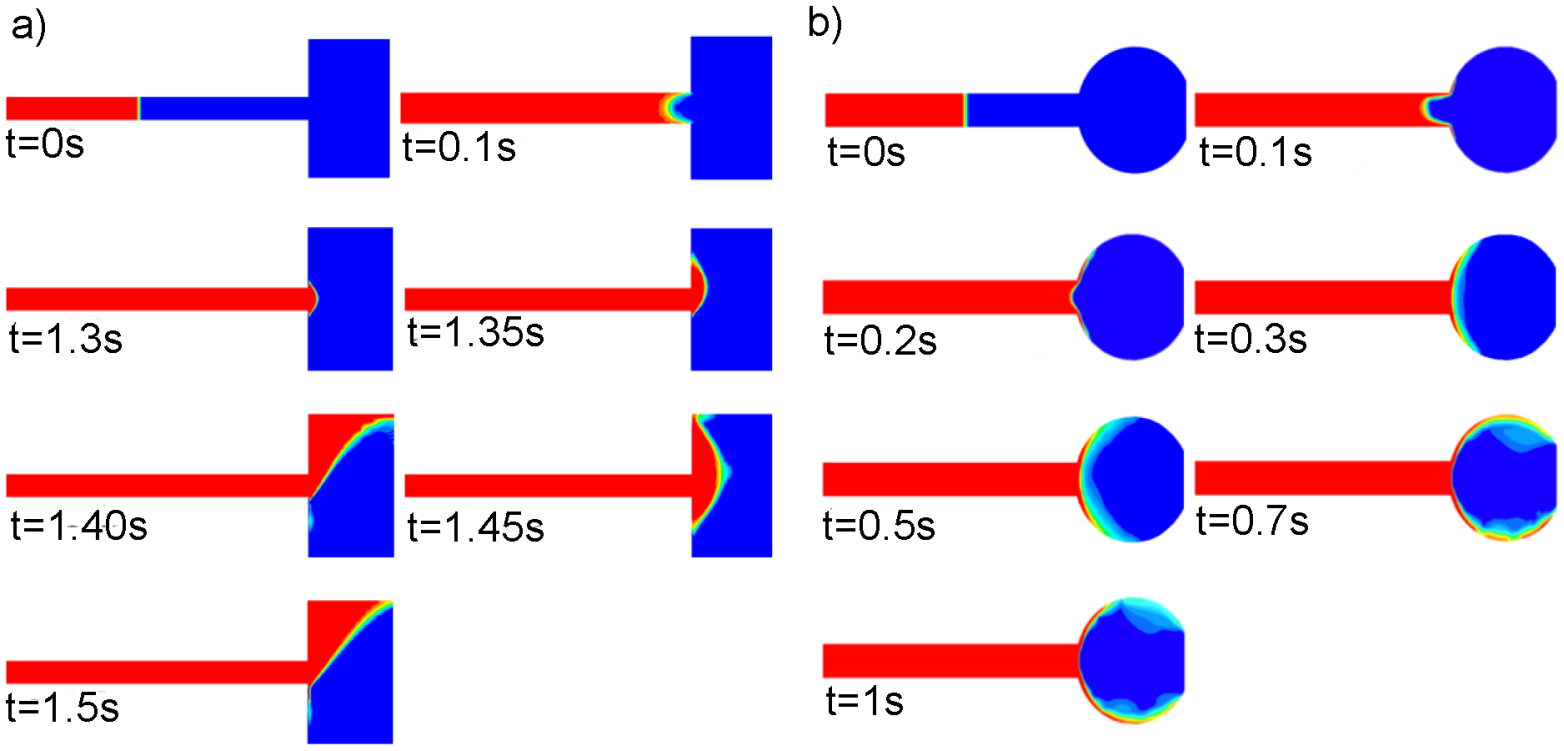
Sequences of the fluid motion in a, a) hydrophilic and b) super hydrophilic capillary.

The difference of flow motion in a hydrophilic and a super hydrophilic capillary is illustrated in Fig. 4. Before the fluid reaches the valve point, i.e., at t = 0.1s and earlier, the meniscus in both capillary has a concave shape due to the hydrophilicity of capillary surfaces. For a hydrophilic capillary with an assumed contact angle of θ  = 60° as shown in Fig 4a, at t = 1.3s due to the capillary pressure barrier, the fluid stops at the very end of the capillary and the meniscus shape is gradually changed into a convex shape. As the rotational speed increases to 275rpm, the centrifugal pressure overcomes the pressure barrier (t = 1.35s) and the fluid bursts into the right chamber. It flows at the top of the chamber against the clockwise rotational direction probably due to the inertial force and the start of Coriolis effect. After t = 1.5s, the fluid consistently flow towards the top the chamber. These observations are confirmed in the experimental studies of Cho et al. [8] and Man et al. [22].

The fluid motion in a super hydrophilic capillary with an assumed contact angle of θ  = 20° (Case 11) is shown in Fig. 4b. At this low a contact angle, on contrary to Fig 4a fluid does not completely stop at the capillary valve. While the meniscus retains its concave shape, the fluid flows on side walls and continuously leaks into the expanded volume (right chamber) at very low burst frequencies (<150rpm). The fluid flows symmetrically at the top and bottom side walls of the circular chamber, and cause the change of the meniscus shape, as shown at t = 0.2s to t = 1s. Differences seen between hydrophilic and super hydrophilic capillaries are due to the significant influence of the adhesive wall force in the case of a low contact angle, which eases the flow over expansion surfaces at a very low rotational speed. Moreover, the Coriolis force is dependent on the angular velocity (eq. 13) therefore, operating at a low rotational speed results in inadequate Coriolis force to effect on the direction of the flow advancement in circular chamber. 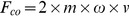
(13)


Similar flow motion to Fig. 4b is also observed for the rectangular structure under the same model set up (the results are not presented here).

The fact that on contrary to the theoretical studies, the numerical data shows the fluid leakage at the low contact angles can be due to several reasons. The theoretical expressions i.e., Eqs.11 and 12 have been verified experimentally for a particular operational parameters e.g., specific geometry of the capillary expansion and specific contact angles i.e., 68°, 70°, 93°. In addition, the theoretical models do not explicitly include the effect of wall adhesion that has a significant influence on fluid interfaces at a low contact angle. However, in the VOF method, the wall adhesion effect is fully considered in the governing equations (see Eq. 9).

### 3.3 Effect of dimensions of the capillary channel

**Figure 5:**
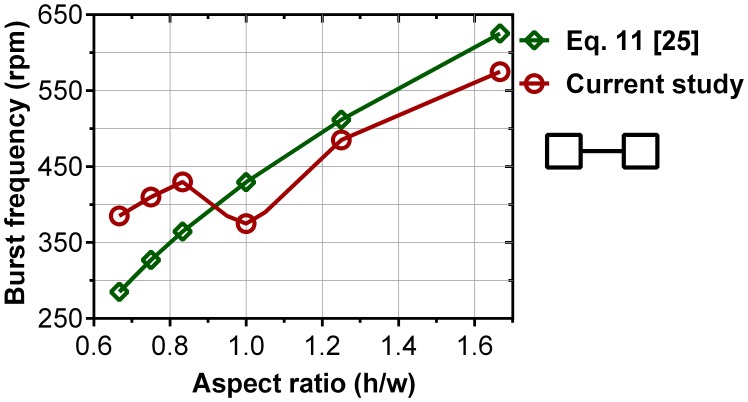
Burst frequency of the CFD model and Eq. 11 [25] versus aspect ratio for Cases 5–10.

Fig. 5 plots the computed burst frequency against the aspect ratio (AR) for Cases 5–10 where with a constant height of 300 µm, the capillary width is varied from 180 µm to 450 µm. The burst frequencies calculated by theoretical model is also plotted for the comparison.

**Figure 6:**
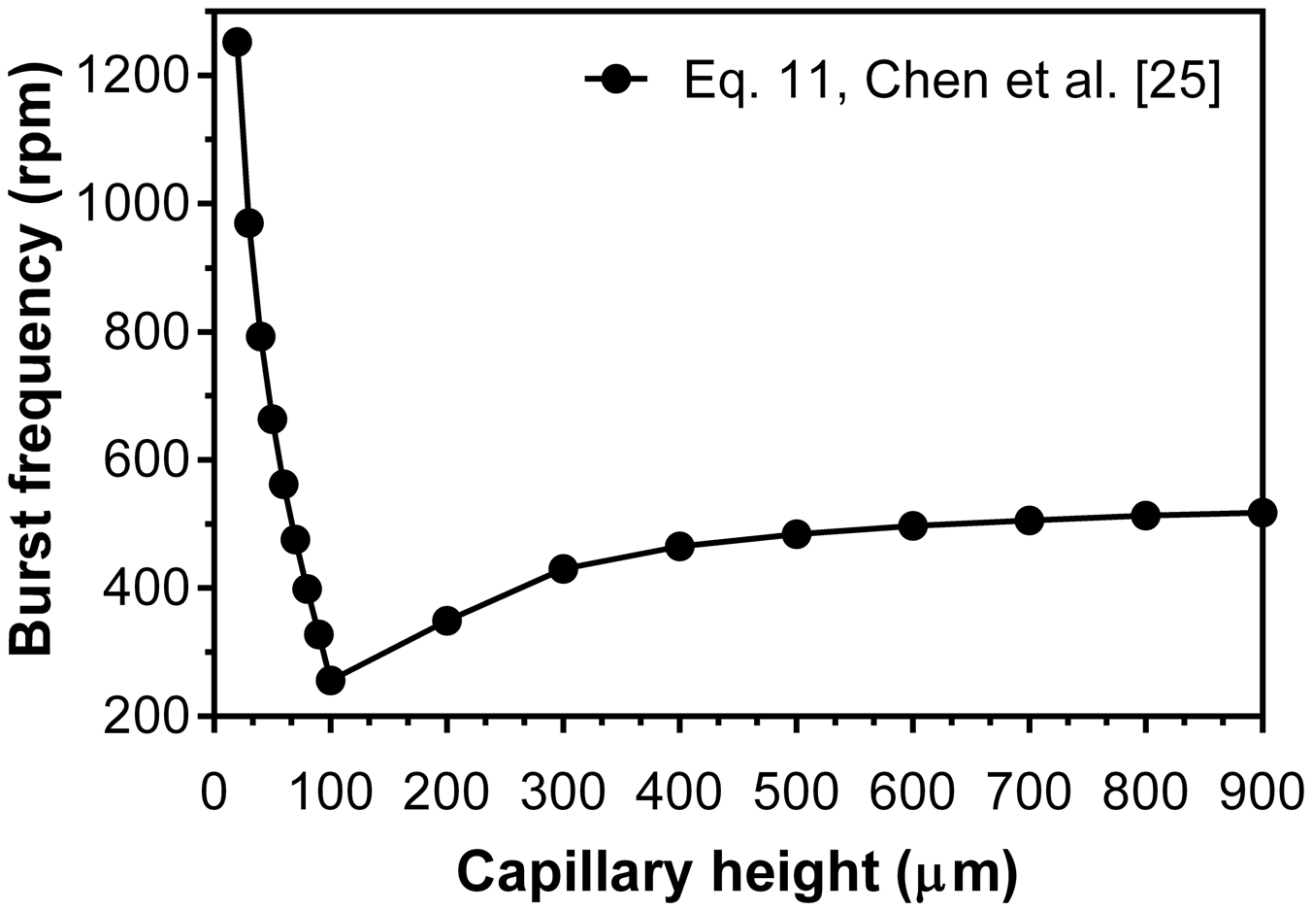
The distribution of the burst frequencies using Eq. 11 [25] versus different capillary height where the capillary width is kept constant of 300 µm for a contact of 70.

The results show that for a constant height (300 µm), burst frequencies increase as the capillary width decreases. It shows similar trend to experiments by Chen et al. [25] where the capillary height varied and width kept constant (300 µm). Therefore, lower burst frequencies are always expected for wide capillaries (w>h) compared to narrow capillaries (h>w). This probably is because of the microfluidics structures used in the present study and that of the theoretical models. In such structures alteration in capillary height and its width do not have the identical effect on the burst frequency. In the width direction the capillary is bounded by solid walls while in its height direction it is not; that results in greater influence of the height on the burst frequencies compared to capillary width (see Fig. 3). The influence of capillary height on burst frequencies calculated from theoretical models are plotted Fig. 6. For a large difference between the capillary height and its width (h/w from 0.06 to 0.33) the burst frequency is highly susceptible to capillary height. For instance, with an increase in capillary height from 10 µm to 100 µm the burst frequency increases from 250rpm to 1200rpm. On the contrary, when there is a small difference between the height and width of the capillary (1<h/w<3) a large increase in height (300 µm to 900 µm) only increases the burst frequency from 430rpm to 525rpm. Note that, the theoretical expressions have been tested merely for conventional capillary dimensions. A possible reason for the contrary trend can be rather large dimension of the capillaries which is not commonly used in centrifugal microfluidic platforms.

The computed results show the minima burst frequencies for square capillaries (AR = 1). The minimum burst frequency in square capillaries can be seen in Fig. 5 where at aspect ratio of 1, our numerical value of the burst frequency is 375rpm. However, these minima are not predicted by previous theoretical models, they are predicted well in the theoretical model we have developed [47]. The lower burst frequency of the square capillaries compared to the rectangular capillaries can be seen in our experimental results listed in Table 3. The experiments were carried out for a constant capillary width of 400 µm and depth of 200 µm, 300 µm, 400 µm and 500 µm to further investigate the burst frequency in square and rectangular capillaries. Table 3 shows that the burst frequency lowers in square capillaries in comparison with the rectangular ones. These minima can be probably due to the specific symmetrical shape of the capillary that reduces the difference between the liquid-air interfaces on both the horizontal and vertical directions of the capillary. In the same manner, the equal distribution of the centrifugal force on the same directions may cause the minima burst frequencies. Therefore, the meniscus will be exposed to a uniform tension that can cause an early burst.

**Table 3:** A comparison between burst frequencies of square and rectangular capillary valves.

Capillary	rˆ	σ_la_	θ°	width	depth	Exp.	Num.
section	(mm)	(N/m)		(µm)	(µm)	burst (rpm)	burst (rpm)
Rectangular	45	0.072	77	400	200	350–370	350–400
Rectangular	45	0.072	77	400	300	300–320	300–350
Square	45	0.072	77	400	400	230–260	200–250
Rectangular	45	0.072	77	400	500	290–310	300–350

### 3.4 Effect of contact angles on burst frequency

**Figure 7:**
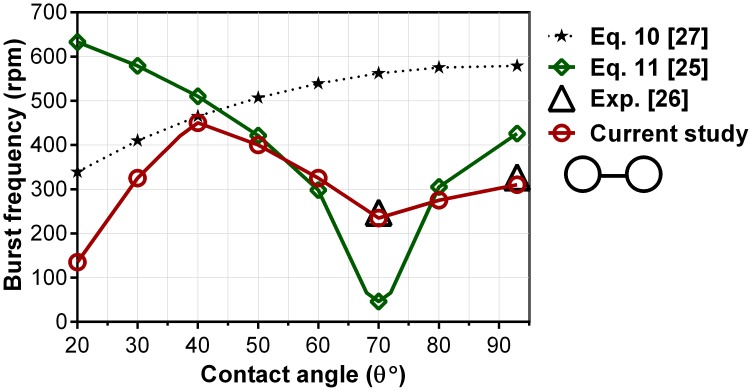
Burst frequency from the CFD model, Eq. 10 [27] & Eq. 11 [25] versus contact angles for Cases 11–18. Experiment results from He et al. [26] is also given.

**Figure 8:**
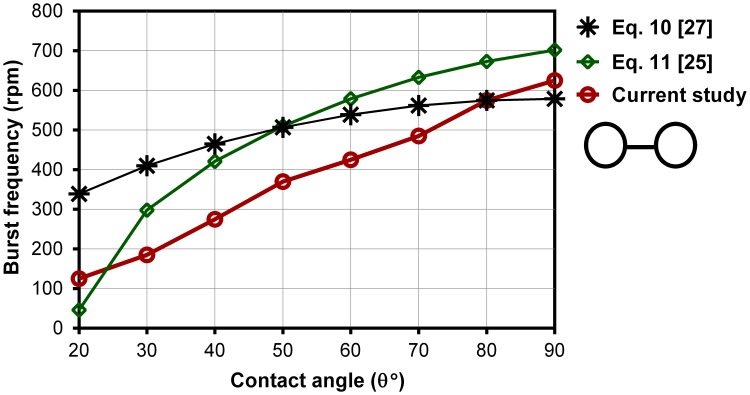
Burst frequencies from the CFD model, Eq. 10 [27] & Eq. 11 [25] versus contact angles for Cases 19–26.

Figs. 7, 8 show the effect of the contact angles on burst frequencies of wide capillaries (w>h) and narrow capillaries, respectively. Fig. 7 contains numerically computed results for Cases 11–18 where the height to width ratio of the capillaries is 157/426. Experimental results from He et al. [26] for θ = 70° and θ = 93° and the theoretical models are included for comparison with the numerical results. The computed results are in excellent agreement with those of experimental from He et al. [26]. They show two lowest burst frequencies which occur at θ = 20° (<150rpm) and at θ = 70° (240 rpm). For super hydrophilic centrifugal microfluidic platforms (θ<40°), a decreasing trend of burst frequency with the decreasing of the contact angle can be expected due the increase of the wall adhesion effect. With a strong adhesion wall force, the fluid leaks even at a small centrifugal force as shown in Fig. 4. For contact angles between 40° and 90°, first the burst frequency decreases with increasing contact angle until the minimum burst frequency of 240rpm, which occurs at 70° contact angle. This is quite interesting and we regard this minimum point as an optimum contact angle for the channel’s dimensions, i.e., aspect ratio (h/w) of this case. For a different aspect ratio, the minimum burst frequency occurs at a different contact angle. Following the minimum point, the burst frequency only slightly increases with the increase of the contact angle, i.e., from about 250rpm to about 325rpm. The highest burst frequency occurs at the contact angle of 40° (about 450 rpm).

For super hydrophilic centrifugal microfluidic platforms (<40°) numerical results and Eq. 10 both show the increase of burst frequencies with the increase of the contact angle which is in contradiction to Eq. 11. Despite of the high burst frequencies predicted by theoretical model our numerical results show that fluid flows over the capillary expansion walls before it actually bursts. In fact, the fluid leaks into the desired chamber before applying adequate centrifugal pressure that causes the change of the meniscus shape in hydrophilic centrifugal microfluidic platforms (Fig. 4). At the contact angle 70° the significant drop of the burst frequency predicted by theoretical models is on contrary to the experiments from He et al. [26] (∼200rpm difference). This exaggeration in predicting of the burst frequency can be expected for other configurations of capillaries especially when the theoretical models calculate an extremely low burst frequency. Fig. 8 shows the effect of the contact angles on burst frequencies for narrow capillaries (h>w). The height/width ratio is 426/157 which is the opposite of that used in Cases 11–18 (i.e., 157/426). In general, for narrow capillaries (h>w) the numerical results show the increase of burst frequency with the increase of the contact angle. On contrary to wide capillaries (h<w) both the numerical and the theoretical models have the similar trends.

**Figure 9:**
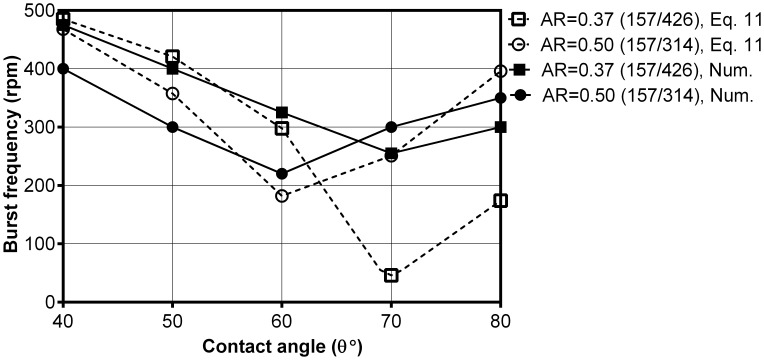
The distribution of the burst frequencies from the CFD model and Eq. 11 [25] with respect to different aspect ratios and contact angles for Cases 27–42.

Fig. 9 shows the distribution of the computed burst frequencies for different capillary dimensions versus the contact angles (Cases 27–36). Herein, Geo. 1 has been used instead of Geo. 2 (used in Fig. 7) to extend our discussion about the minima burst frequencies. Similar to Fig. 7 computed results show a minimum burst frequency which changes with the capillary dimensions. Although theoretical models almost successfully predict such minima, the values calculate by these models can be extremely different than those of experiments. These values are considered too small for burst frequencies in any centrifugal microfluidic platforms. The burst frequency of most of the cases in the literature is above 250rpm with very limited cases below 250rpm [2], [9], [15], [25], [26], [44], [48]–[51].

## Conclusions

Flow in passive capillary microvalves in centrifugal microfluidic platforms for a range of superhydrophilic to less hydrophilic contact angles has been studied using the VOF model within the commercial CFD code of ANSYS Fluent. Our experimental data in addition to experiment results from Chen et al. [25], He et al. [26] and Gliere et al. [44] were used for validation and computed results were compared with existing theoretical models. The findings of the current study can be summarized as:

- In common capillary dimensions (>100 µm) for the cases of a low contact angle especially less than 20°, the capillary valve is unable to retain the fluid from leaking and it loses its function.
- The computed results suggest that the theoretical models cannot be used for super hydrophilic materials since they are unable to predict the fluid leakage. While they predict that high pressure is required for pushing the fluid over capillary valves, the computed results show that fluid flows consistently over the capillary valve into the next chamber at low pressures.
- In general, computed results show that burst frequencies of wide capillaries (w>h) are always lower than those of narrow capillaries (w<h). Theoretical models predict similar to our computed results for wide and narrow capillaries.
- The computed results for narrow capillaries (w<h) show a consistent increase of burst frequencies with the increase of the contact angle. However, for wide capillaries (w>h) the computed results predict three divisions of burst frequencies. First, as the contact angle increases, the burst frequency increases to a peak where it starts decreasing at a low burst frequency. After a low burst frequency it slightly increases with the increase of the contact angle.
- The results show that burst frequencies of square capillaries are lower than those of rectangular shapes. However, the theoretical models used for comparison are not able to predict pressure drops in square capillaries; our experimental data and the theoretical model that we have developed [47] show this pressure drops.
